# *N*′-[(1*E*)-(2-Hy­droxy­naphthalen-1-yl)methyl­idene]-2-meth­oxy­benzohydrazide

**DOI:** 10.1107/S1600536811038153

**Published:** 2011-09-30

**Authors:** Xiao-Yan Li

**Affiliations:** a Zibo Vocational Institute, Zibo 255314, People’s Republic of China

## Abstract

There are three independent mol­ecules in the asymmetric unit of the title compound, C_19_H_16_N_2_O_3_, in which the dihedral angles between the naphthalene ring system and the benzene ring are 7.52 (16), 18.15 (18), and 13.9 (2)°. All the mol­ecules exist in the *trans* configuration with respect to the methyl­idene units. In each mol­ecule there is one O—H⋯N and one N—H⋯O intra­molecular hydrogen bond. In the crystal, two of the mol­ecules are linked *via* a bifurcated N—H⋯(O,O) hydrogen bond. All three mol­ecules are further linked *via* C—H⋯O inter­actions.

## Related literature

For the syntheses and crystal structures of hydrazone compounds, see: Hashemian *et al.* (2011 ▶); Lei (2011 ▶); Shalash *et al.* (2010 ▶); Li (2011*a* ▶). For the crystal structures of similar compounds reported recently by the author, see: Li (2011*b* ▶,*c* ▶).
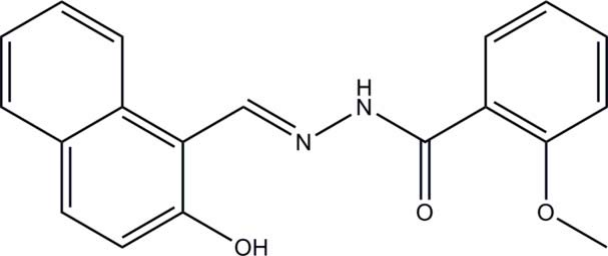


## Experimental

### 

#### Crystal data

C_19_H_16_N_2_O_3_
*M* *_r_* = 320.34
Triclinic, 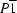

*a* = 12.2266 (19) Å
*b* = 14.212 (3) Å
*c* = 15.805 (2) Å
α = 107.507 (3)°
β = 108.475 (3)°
γ = 95.885 (3)°
*V* = 2423.9 (7) Å^3^
*Z* = 6
Mo *K*α radiation
μ = 0.09 mm^−1^
*T* = 298 K
0.23 × 0.23 × 0.21 mm

#### Data collection

Bruker SMART CCD area-detector diffractometer
Absorption correction: multi-scan (*SADABS*; Sheldrick, 1996 ▶) *T*_min_ = 0.980, *T*_max_ = 0.981
20059 measured reflections
10353 independent reflections
3438 reflections with *I* > 2σ(*I*)
*R*_int_ = 0.054

#### Refinement

*R*[*F* ^2^ > 2σ(*F* ^2^)] = 0.061
*wR*(*F* ^2^) = 0.184
*S* = 0.94
10353 reflections
666 parameters
4 restraints
H atoms treated by a mixture of independent and constrained refinement
Δρ_max_ = 0.13 e Å^−3^
Δρ_min_ = −0.15 e Å^−3^

### 

Data collection: *SMART* (Bruker, 1998 ▶); cell refinement: *SAINT* (Bruker, 1998 ▶); data reduction: *SAINT*; program(s) used to solve structure: *SHELXS97* (Sheldrick, 2008 ▶); program(s) used to refine structure: *SHELXL97* (Sheldrick, 2008 ▶); molecular graphics: *SHELXTL* (Sheldrick, 2008 ▶); software used to prepare material for publication: *SHELXTL*.

## Supplementary Material

Crystal structure: contains datablock(s) global, I. DOI: 10.1107/S1600536811038153/su2316sup1.cif

Structure factors: contains datablock(s) I. DOI: 10.1107/S1600536811038153/su2316Isup2.hkl

Supplementary material file. DOI: 10.1107/S1600536811038153/su2316Isup3.cml

Additional supplementary materials:  crystallographic information; 3D view; checkCIF report

## Figures and Tables

**Table 1:** Hydrogen-bond geometry (Å, °)

*D*—H⋯*A*	*D*—H	H⋯*A*	*D*⋯*A*	*D*—H⋯*A*
O1—H1⋯N1	0.82	1.84	2.556 (3)	145
N2—H2⋯O3	0.91 (1)	1.94 (2)	2.622 (3)	131 (3)
N2—H2⋯O8^i^	0.91 (1)	2.41 (2)	3.094 (4)	132 (2)
O4—H4⋯N3	0.86 (1)	1.82 (2)	2.538 (4)	141 (3)
N4—H4*B*⋯O6	0.91 (1)	1.95 (2)	2.647 (4)	132 (3)
O7—H7⋯N5	0.82	1.80	2.517 (4)	146
N6—H6*A*⋯O9	0.89 (1)	1.89 (2)	2.621 (4)	138 (3)
C11—H11⋯O8^i^	0.93	2.46	3.122 (4)	129
C38—H38*B*⋯O2^ii^	0.96	2.35	3.090 (5)	133
C57—H57*B*⋯O4^iii^	0.96	2.56	3.435 (5)	151

Symmetry codes: (i) 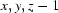; (ii) 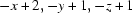; (iii) 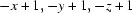.

**Fig. 1.**
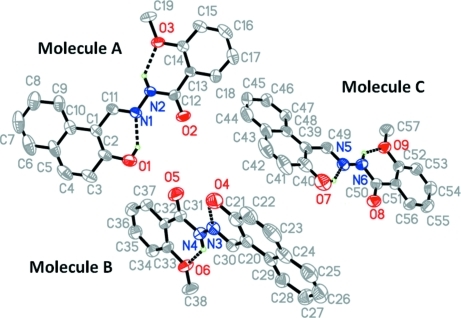
The molecular structure of the three independent molecules (A, B and C) of the title compound, showing the atom labelling. The displacement ellipsoids are drawn at the 30% probability level. Intramolecular O-H···N and N-H···O hydrogen bonds are indicated by dashed lines. The C-bound H-atoms have been omitted for clarity.

## supplementary crystallographic information

### Comment

In the last few years, hydrazones have attracted much attention for their syntheses and crystal structures (Hashemian *et al.*, 2011; Lei, 2011; Shalash *et al.*, 2010). As a continuation of our work on such compounds (Li, 2011*a*,*b*), the author reports herein on the crystal structure of the new title hydrazone compound.

In the asymmetric unit of the title compound there are three (A, B and C) independent molecules (Fig. 1). The bond distances and angles are comparable to those observed in similar compounds (Li, 2011*b*,*c*). The dihedral angles between the naphthalene and the benzene rings in the three molecules are 7.52 (16), 18.15 (18), and 13.9 (2)°, for molecules A, B and C, respectively. All the molecules exist in the *trans* configuration with respect to the methylidene units. In each molecule there is an O-H···N and a N-H···O intramolecular hydrogen bond (Table 1).

In the crystal the A and C molecules are linked by a bifurcated hydrogen bond involving the NH group of molecule A [N2-H2···O8] (Table 1). All three molecules are further linked to one another via C-H···O interactions (Table 1).

### Experimental

A mixture of 2-methoxybenzhydrazide (0.166 g, 1 mmol) and 2-hydroxy-1-naphthaldehyde (0.172 g, 1 mmol) in 30 ml of ethanol containing few drops of acetic acid was refluxed for about 1 h. On cooling to room temperature, a solid precipitate was formed. The solid was filtered and then recrystallized from methanol. Colourless crystals, suitable for X-ray diffraction analysis, were obtained by slow evaporation of a solution of the title compound in methanol.

### Refinement

The NH H-atoms were located from a difference Fourier map and were freely refined. The OH and C-bound H-atoms were positioned geometrically and refined using a riding model: O—H = 0.82 Å, C—H = 0.93 and 0.96 Å, for CH and CH_3_ H-atoms, respectively, with U_iso_(H) = k × U_eq_(O,C), where k = 1.5 for OH and CH_3_ H-atoms, and k = 1.2 for all other H-atoms.

### Crystal data

**Table:**

C_19_H_16_N_2_O_3_	*Z* = 6
*M**_r_* = 320.34	*F*(000) = 1008
Triclinic, *P*1	*D*_x_ = 1.317 Mg m^−^^3^
*a* = 12.2266 (19) Å	Mo *K*α radiation, λ = 0.71073 Å
*b* = 14.212 (3) Å	Cell parameters from 1972 reflections
*c* = 15.805 (2) Å	θ = 2.7–25.0°
α = 107.507 (3)°	µ = 0.09 mm^−^^1^
β = 108.475 (3)°	*T* = 298 K
γ = 95.885 (3)°	Block, colourless
*V* = 2423.9 (7) Å^3^	0.23 × 0.23 × 0.21 mm

### Data collection

**Table:**

Bruker SMART CCD area-detector diffractometer	10353 independent reflections
Radiation source: fine-focus sealed tube	3438 reflections with *I* > 2σ(*I*)
graphite	*R*_int_ = 0.054
ω scans	θ_max_ = 27.0°, θ_min_ = 2.3°
Absorption correction: multi-scan (*SADABS*; Sheldrick, 1996)	*h* = −15→15
*T*_min_ = 0.980, *T*_max_ = 0.981	*k* = −18→17
20059 measured reflections	*l* = −20→20

### Refinement

**Table:**

Refinement on *F*^2^	Primary atom site location: structure-invariant direct methods
Least-squares matrix: full	Secondary atom site location: difference Fourier map
*R*[*F*^2^ > 2σ(*F*^2^)] = 0.061	Hydrogen site location: inferred from neighbouring sites
*wR*(*F*^2^) = 0.184	H atoms treated by a mixture of independent and constrained refinement
*S* = 0.94	*w* = 1/[σ^2^(*F*_o_^2^) + (0.0526*P*)^2^] where *P* = (*F*_o_^2^ + 2*F*_c_^2^)/3
10353 reflections	(Δ/σ)_max_ < 0.001
666 parameters	Δρ_max_ = 0.13 e Å^−^^3^
4 restraints	Δρ_min_ = −0.15 e Å^−^^3^

### Special details

**Table:**

Geometry. All esds (except the esd in the dihedral angle between two l.s. planes) are estimated using the full covariance matrix. The cell esds are taken into account individually in the estimation of esds in distances, angles and torsion angles; correlations between esds in cell parameters are only used when they are defined by crystal symmetry. An approximate (isotropic) treatment of cell esds is used for estimating esds involving l.s. planes.
Refinement. Refinement of F^2^ against ALL reflections. The weighted R-factor wR and goodness of fit S are based on F^2^, conventional R-factors R are based on F, with F set to zero for negative F^2^. The threshold expression of F^2^ > 2sigma(F^2^) is used only for calculating R-factors(gt) etc. and is not relevant to the choice of reflections for refinement. R-factors based on F^2^ are statistically about twice as large as those based on F, and R- factors based on ALL data will be even larger.

### Fractional atomic coordinates and isotropic or equivalent isotropic displacement parameters (Å^2^)

**Table:**

	*x*	*y*	*z*	*U*_iso_*/*U*_eq_	
N1	0.7028 (2)	0.66822 (19)	0.12093 (18)	0.0611 (7)	
N2	0.5820 (2)	0.6409 (2)	0.09232 (18)	0.0600 (7)	
N3	0.9007 (2)	0.1667 (2)	0.4186 (2)	0.0708 (8)	
N4	0.9645 (3)	0.2611 (2)	0.43764 (19)	0.0687 (8)	
N5	0.4514 (2)	0.7856 (2)	0.8040 (2)	0.0738 (8)	
N6	0.4112 (3)	0.8381 (2)	0.8720 (2)	0.0740 (8)	
O1	0.9121 (2)	0.6929 (2)	0.23860 (18)	0.0873 (8)	
H1	0.8399	0.6837	0.2209	0.131*	
O2	0.6032 (2)	0.60244 (19)	0.22336 (17)	0.0933 (8)	
O3	0.36430 (18)	0.60654 (17)	−0.02694 (15)	0.0761 (7)	
O4	0.7731 (2)	−0.0019 (2)	0.2966 (2)	0.1034 (9)	
O5	0.8678 (2)	0.2523 (2)	0.28836 (18)	0.0987 (9)	
O6	1.1456 (2)	0.41689 (17)	0.54154 (16)	0.0831 (7)	
O7	0.5615 (3)	0.6551 (2)	0.7471 (3)	0.1237 (11)	
H7	0.5334	0.6886	0.7837	0.186*	
O8	0.5332 (2)	0.78589 (19)	0.97944 (17)	0.0900 (8)	
O9	0.2894 (2)	0.96365 (18)	0.93681 (17)	0.0842 (7)	
C1	0.8710 (3)	0.7184 (2)	0.0879 (3)	0.0618 (9)	
C2	0.9480 (3)	0.7153 (2)	0.1729 (3)	0.0691 (10)	
C3	1.0712 (3)	0.7350 (3)	0.1956 (3)	0.0917 (13)	
H3	1.1215	0.7328	0.2528	0.110*	
C4	1.1155 (4)	0.7569 (3)	0.1350 (4)	0.1004 (15)	
H4A	1.1970	0.7697	0.1512	0.120*	
C5	1.0438 (4)	0.7614 (3)	0.0477 (4)	0.0866 (12)	
C6	1.0888 (5)	0.7824 (3)	−0.0165 (5)	0.124 (2)	
H6	1.1704	0.7967	0.0004	0.149*	
C7	1.0206 (6)	0.7832 (4)	−0.1024 (5)	0.133 (2)	
H7A	1.0542	0.7956	−0.1443	0.160*	
C8	0.8966 (5)	0.7646 (3)	−0.1265 (3)	0.1155 (16)	
H8	0.8478	0.7661	−0.1845	0.139*	
C9	0.8477 (4)	0.7444 (3)	−0.0652 (3)	0.0891 (12)	
H9	0.7660	0.7319	−0.0826	0.107*	
C10	0.9190 (4)	0.7421 (2)	0.0238 (3)	0.0732 (10)	
C11	0.7438 (3)	0.6925 (2)	0.0634 (2)	0.0609 (9)	
H11	0.6925	0.6935	0.0061	0.073*	
C12	0.5386 (3)	0.6067 (2)	0.1492 (2)	0.0607 (9)	
C13	0.4076 (3)	0.5741 (2)	0.1165 (2)	0.0538 (8)	
C14	0.3236 (3)	0.5751 (2)	0.0337 (2)	0.0594 (9)	
C15	0.2043 (3)	0.5470 (3)	0.0149 (3)	0.0763 (10)	
H15	0.1487	0.5505	−0.0394	0.092*	
C16	0.1684 (3)	0.5137 (3)	0.0771 (4)	0.0897 (12)	
H16	0.0882	0.4938	0.0642	0.108*	
C17	0.2497 (4)	0.5097 (3)	0.1577 (3)	0.0898 (12)	
H17	0.2249	0.4869	0.1993	0.108*	
C18	0.3668 (3)	0.5392 (2)	0.1769 (2)	0.0703 (10)	
H18	0.4213	0.5360	0.2318	0.084*	
C19	0.2841 (3)	0.5996 (3)	−0.1172 (2)	0.0881 (12)	
H19A	0.2358	0.5324	−0.1498	0.132*	
H19B	0.3275	0.6145	−0.1544	0.132*	
H19C	0.2347	0.6472	−0.1084	0.132*	
C20	0.8568 (3)	0.0289 (3)	0.4651 (3)	0.0623 (9)	
C21	0.7909 (3)	−0.0327 (4)	0.3714 (3)	0.0811 (11)	
C22	0.7382 (4)	−0.1347 (4)	0.3495 (3)	0.1021 (14)	
H22	0.6970	−0.1763	0.2863	0.123*	
C23	0.7474 (4)	−0.1719 (3)	0.4197 (4)	0.1044 (15)	
H23	0.7119	−0.2391	0.4040	0.125*	
C24	0.8093 (3)	−0.1120 (3)	0.5166 (4)	0.0821 (11)	
C25	0.8153 (4)	−0.1510 (4)	0.5892 (5)	0.1082 (16)	
H25	0.7780	−0.2177	0.5735	0.130*	
C26	0.8746 (5)	−0.0932 (4)	0.6814 (5)	0.1133 (16)	
H26	0.8771	−0.1195	0.7291	0.136*	
C27	0.9322 (4)	0.0064 (4)	0.7052 (3)	0.0955 (13)	
H27	0.9744	0.0461	0.7688	0.115*	
C28	0.9269 (3)	0.0457 (3)	0.6359 (3)	0.0769 (10)	
H28	0.9652	0.1124	0.6533	0.092*	
C29	0.8659 (3)	−0.0110 (3)	0.5396 (3)	0.0660 (9)	
C30	0.9174 (3)	0.1300 (3)	0.4855 (3)	0.0672 (10)	
H30	0.9686	0.1688	0.5471	0.081*	
C31	0.9472 (3)	0.2980 (3)	0.3656 (3)	0.0710 (10)	
C32	1.0257 (3)	0.3946 (3)	0.3839 (2)	0.0610 (9)	
C33	1.1207 (3)	0.4525 (3)	0.4678 (2)	0.0647 (9)	
C34	1.1860 (3)	0.5412 (3)	0.4743 (3)	0.0800 (11)	
H34	1.2493	0.5787	0.5303	0.096*	
C35	1.1584 (4)	0.5745 (3)	0.3986 (3)	0.0912 (12)	
H35	1.2022	0.6347	0.4036	0.109*	
C36	1.0657 (4)	0.5184 (3)	0.3157 (3)	0.0909 (12)	
H36	1.0467	0.5406	0.2642	0.109*	
C37	1.0008 (3)	0.4294 (3)	0.3083 (2)	0.0777 (11)	
H37	0.9388	0.3918	0.2514	0.093*	
C38	1.2370 (3)	0.4773 (3)	0.6314 (2)	0.0971 (13)	
H38A	1.3115	0.4855	0.6232	0.146*	
H38B	1.2412	0.4442	0.6768	0.146*	
H38C	1.2194	0.5424	0.6543	0.146*	
C39	0.4664 (3)	0.7488 (3)	0.6523 (3)	0.0709 (10)	
C40	0.5324 (4)	0.6785 (3)	0.6673 (4)	0.0955 (13)	
C41	0.5746 (5)	0.6247 (4)	0.5978 (5)	0.128 (2)	
H41	0.6186	0.5770	0.6089	0.154*	
C42	0.5511 (5)	0.6422 (4)	0.5161 (5)	0.131 (2)	
H42	0.5785	0.6056	0.4705	0.157*	
C43	0.4857 (4)	0.7148 (4)	0.4970 (4)	0.0979 (14)	
C44	0.4633 (5)	0.7349 (5)	0.4128 (4)	0.134 (2)	
H44	0.4898	0.6978	0.3668	0.161*	
C45	0.4043 (5)	0.8068 (6)	0.3963 (4)	0.144 (2)	
H45	0.3892	0.8184	0.3392	0.173*	
C46	0.3663 (4)	0.8635 (4)	0.4661 (4)	0.1291 (19)	
H46	0.3280	0.9147	0.4563	0.155*	
C47	0.3847 (3)	0.8446 (4)	0.5489 (3)	0.0974 (13)	
H47	0.3570	0.8820	0.5938	0.117*	
C48	0.4444 (3)	0.7701 (3)	0.5669 (3)	0.0792 (11)	
C49	0.4215 (3)	0.8000 (3)	0.7241 (3)	0.0731 (10)	
H49	0.3710	0.8431	0.7123	0.088*	
C50	0.4587 (3)	0.8365 (3)	0.9618 (3)	0.0715 (10)	
C51	0.4183 (3)	0.8957 (3)	1.0363 (3)	0.0700 (10)	
C52	0.3376 (3)	0.9574 (3)	1.0247 (3)	0.0717 (10)	
C53	0.3071 (3)	1.0091 (3)	1.1009 (3)	0.0893 (12)	
H53	0.2534	1.0505	1.0931	0.107*	
C54	0.3569 (4)	0.9986 (3)	1.1876 (3)	0.1009 (14)	
H54	0.3354	1.0322	1.2382	0.121*	
C55	0.4380 (4)	0.9392 (4)	1.2006 (3)	0.1055 (15)	
H55	0.4720	0.9330	1.2598	0.127*	
C56	0.4681 (3)	0.8889 (3)	1.1252 (3)	0.0915 (12)	
H56	0.5236	0.8492	1.1343	0.110*	
C57	0.2108 (4)	1.0304 (3)	0.9225 (3)	0.1040 (14)	
H57A	0.1420	1.0096	0.9350	0.156*	
H57B	0.1874	1.0282	0.8578	0.156*	
H57C	0.2502	1.0982	0.9652	0.156*	
H2	0.534 (2)	0.648 (2)	0.0388 (13)	0.080*	
H4B	1.022 (2)	0.296 (2)	0.4955 (12)	0.080*	
H6A	0.360 (2)	0.878 (2)	0.864 (2)	0.080*	
H4	0.805 (3)	0.0608 (10)	0.314 (2)	0.080*	

### Atomic displacement parameters (Å^2^)

**Table:**

	*U*^11^	*U*^22^	*U*^33^	*U*^12^	*U*^13^	*U*^23^
N1	0.0485 (18)	0.0647 (19)	0.0694 (19)	0.0107 (14)	0.0166 (15)	0.0283 (16)
N2	0.0452 (19)	0.074 (2)	0.0614 (18)	0.0125 (15)	0.0121 (15)	0.0332 (17)
N3	0.0604 (19)	0.079 (2)	0.077 (2)	0.0174 (17)	0.0270 (16)	0.0291 (19)
N4	0.069 (2)	0.079 (2)	0.0596 (19)	0.0171 (18)	0.0200 (15)	0.0288 (19)
N5	0.069 (2)	0.068 (2)	0.086 (2)	0.0205 (16)	0.0315 (18)	0.0263 (19)
N6	0.074 (2)	0.079 (2)	0.079 (2)	0.0285 (17)	0.0248 (19)	0.040 (2)
O1	0.0634 (17)	0.100 (2)	0.0949 (19)	0.0151 (17)	0.0149 (15)	0.0453 (17)
O2	0.0706 (17)	0.129 (2)	0.0769 (17)	0.0049 (16)	0.0023 (14)	0.0630 (17)
O3	0.0570 (15)	0.0951 (19)	0.0648 (15)	0.0035 (13)	0.0030 (12)	0.0374 (14)
O4	0.082 (2)	0.121 (3)	0.0783 (19)	0.0030 (19)	0.0182 (16)	0.014 (2)
O5	0.0836 (19)	0.124 (2)	0.0684 (17)	−0.0067 (17)	0.0066 (15)	0.0385 (17)
O6	0.0902 (18)	0.0897 (19)	0.0557 (15)	0.0138 (15)	0.0062 (13)	0.0305 (14)
O7	0.120 (3)	0.103 (3)	0.181 (3)	0.058 (2)	0.066 (2)	0.074 (3)
O8	0.0828 (19)	0.095 (2)	0.106 (2)	0.0354 (16)	0.0286 (15)	0.0549 (17)
O9	0.0846 (18)	0.092 (2)	0.0844 (19)	0.0358 (15)	0.0275 (14)	0.0402 (16)
C1	0.057 (2)	0.047 (2)	0.079 (3)	0.0125 (18)	0.028 (2)	0.0156 (19)
C2	0.050 (2)	0.053 (2)	0.091 (3)	0.0054 (18)	0.017 (2)	0.017 (2)
C3	0.055 (3)	0.079 (3)	0.120 (4)	0.011 (2)	0.020 (3)	0.020 (3)
C4	0.063 (3)	0.069 (3)	0.147 (5)	0.005 (2)	0.042 (3)	0.008 (3)
C5	0.084 (3)	0.050 (2)	0.134 (4)	0.004 (2)	0.070 (3)	0.015 (3)
C6	0.128 (5)	0.064 (3)	0.191 (6)	−0.004 (3)	0.110 (5)	0.011 (4)
C7	0.188 (7)	0.077 (4)	0.173 (6)	0.009 (4)	0.137 (6)	0.029 (4)
C8	0.165 (5)	0.089 (3)	0.122 (4)	0.021 (3)	0.086 (4)	0.044 (3)
C9	0.105 (3)	0.070 (3)	0.107 (3)	0.006 (2)	0.061 (3)	0.032 (3)
C10	0.078 (3)	0.051 (2)	0.095 (3)	0.011 (2)	0.046 (2)	0.016 (2)
C11	0.055 (2)	0.055 (2)	0.073 (2)	0.0163 (18)	0.0207 (19)	0.0246 (19)
C12	0.060 (2)	0.062 (2)	0.056 (2)	0.0113 (19)	0.0129 (19)	0.0262 (19)
C13	0.048 (2)	0.055 (2)	0.054 (2)	0.0076 (16)	0.0141 (17)	0.0187 (17)
C14	0.055 (2)	0.053 (2)	0.067 (2)	0.0122 (18)	0.0198 (19)	0.0198 (19)
C15	0.057 (3)	0.068 (3)	0.095 (3)	0.014 (2)	0.020 (2)	0.026 (2)
C16	0.056 (3)	0.070 (3)	0.134 (4)	0.010 (2)	0.039 (3)	0.022 (3)
C17	0.090 (3)	0.084 (3)	0.111 (4)	0.013 (3)	0.061 (3)	0.033 (3)
C18	0.075 (3)	0.069 (3)	0.073 (2)	0.014 (2)	0.033 (2)	0.028 (2)
C19	0.085 (3)	0.088 (3)	0.058 (2)	0.009 (2)	−0.010 (2)	0.021 (2)
C20	0.049 (2)	0.065 (3)	0.066 (2)	0.0104 (19)	0.0232 (19)	0.013 (2)
C21	0.062 (3)	0.092 (4)	0.084 (3)	0.014 (2)	0.029 (2)	0.022 (3)
C22	0.074 (3)	0.088 (4)	0.110 (4)	−0.004 (3)	0.032 (3)	−0.004 (3)
C23	0.072 (3)	0.067 (3)	0.160 (5)	0.003 (2)	0.047 (3)	0.020 (4)
C24	0.056 (2)	0.065 (3)	0.123 (4)	0.012 (2)	0.037 (3)	0.027 (3)
C25	0.091 (4)	0.085 (4)	0.180 (5)	0.024 (3)	0.066 (4)	0.070 (4)
C26	0.117 (4)	0.108 (5)	0.165 (5)	0.044 (3)	0.077 (4)	0.084 (4)
C27	0.106 (3)	0.098 (4)	0.106 (3)	0.038 (3)	0.043 (3)	0.058 (3)
C28	0.085 (3)	0.075 (3)	0.081 (3)	0.025 (2)	0.033 (2)	0.035 (3)
C29	0.056 (2)	0.061 (3)	0.092 (3)	0.019 (2)	0.038 (2)	0.030 (2)
C30	0.060 (2)	0.077 (3)	0.067 (2)	0.020 (2)	0.0255 (19)	0.027 (2)
C31	0.064 (3)	0.095 (3)	0.060 (2)	0.025 (2)	0.021 (2)	0.035 (2)
C32	0.060 (2)	0.077 (3)	0.052 (2)	0.021 (2)	0.0194 (19)	0.028 (2)
C33	0.069 (2)	0.078 (3)	0.057 (2)	0.030 (2)	0.024 (2)	0.031 (2)
C34	0.074 (3)	0.091 (3)	0.072 (3)	0.013 (2)	0.020 (2)	0.033 (2)
C35	0.085 (3)	0.097 (3)	0.094 (3)	0.007 (3)	0.031 (3)	0.042 (3)
C36	0.091 (3)	0.108 (4)	0.089 (3)	0.023 (3)	0.034 (3)	0.055 (3)
C37	0.072 (3)	0.103 (3)	0.064 (2)	0.021 (2)	0.021 (2)	0.042 (2)
C38	0.094 (3)	0.118 (3)	0.052 (2)	0.027 (3)	0.000 (2)	0.021 (2)
C39	0.055 (2)	0.058 (3)	0.093 (3)	0.0151 (19)	0.030 (2)	0.014 (2)
C40	0.079 (3)	0.071 (3)	0.135 (4)	0.018 (3)	0.045 (3)	0.027 (3)
C41	0.118 (4)	0.073 (3)	0.208 (7)	0.031 (3)	0.100 (5)	0.022 (4)
C42	0.118 (5)	0.083 (4)	0.175 (6)	0.000 (3)	0.094 (5)	−0.012 (4)
C43	0.071 (3)	0.102 (4)	0.095 (4)	−0.003 (3)	0.045 (3)	−0.009 (3)
C44	0.085 (4)	0.184 (6)	0.095 (4)	−0.016 (4)	0.047 (4)	−0.004 (4)
C45	0.089 (4)	0.254 (8)	0.090 (4)	0.016 (4)	0.044 (3)	0.061 (5)
C46	0.093 (3)	0.226 (6)	0.115 (4)	0.066 (4)	0.057 (3)	0.094 (4)
C47	0.078 (3)	0.148 (4)	0.089 (3)	0.044 (3)	0.039 (2)	0.058 (3)
C48	0.049 (2)	0.099 (3)	0.074 (3)	0.003 (2)	0.020 (2)	0.016 (3)
C49	0.065 (2)	0.071 (3)	0.085 (3)	0.013 (2)	0.023 (2)	0.033 (2)
C50	0.061 (3)	0.064 (3)	0.089 (3)	0.005 (2)	0.019 (2)	0.039 (2)
C51	0.069 (3)	0.067 (3)	0.074 (3)	0.010 (2)	0.019 (2)	0.035 (2)
C52	0.070 (3)	0.065 (3)	0.075 (3)	0.003 (2)	0.022 (2)	0.026 (2)
C53	0.086 (3)	0.083 (3)	0.086 (3)	0.003 (2)	0.023 (3)	0.026 (3)
C54	0.106 (4)	0.095 (4)	0.088 (4)	−0.001 (3)	0.037 (3)	0.018 (3)
C55	0.114 (4)	0.112 (4)	0.081 (3)	0.001 (3)	0.023 (3)	0.043 (3)
C56	0.088 (3)	0.098 (3)	0.087 (3)	0.011 (2)	0.021 (3)	0.045 (3)
C57	0.104 (3)	0.115 (4)	0.115 (3)	0.063 (3)	0.044 (3)	0.054 (3)

### Geometric parameters (Å, °)

**Table:**

N1—C11	1.280 (3)	C21—C22	1.416 (5)
N1—N2	1.375 (3)	C22—C23	1.346 (5)
N2—C12	1.355 (4)	C22—H22	0.9300
N2—H2	0.906 (10)	C23—C24	1.411 (5)
N3—C30	1.283 (4)	C23—H23	0.9300
N3—N4	1.376 (4)	C24—C25	1.403 (5)
N4—C31	1.359 (4)	C24—C29	1.412 (5)
N4—H4B	0.906 (10)	C25—C26	1.346 (6)
N5—C49	1.283 (4)	C25—H25	0.9300
N5—N6	1.371 (4)	C26—C27	1.396 (6)
N6—C50	1.361 (4)	C26—H26	0.9300
N6—H6A	0.887 (10)	C27—C28	1.358 (4)
O1—C2	1.354 (4)	C27—H27	0.9300
O1—H1	0.8200	C28—C29	1.394 (4)
O2—C12	1.212 (3)	C28—H28	0.9300
O3—C14	1.371 (3)	C30—H30	0.9300
O3—C19	1.420 (3)	C31—C32	1.485 (5)
O4—C21	1.343 (4)	C32—C37	1.385 (4)
O4—H4	0.857 (10)	C32—C33	1.399 (4)
O5—C31	1.223 (4)	C33—C34	1.378 (4)
O6—C33	1.367 (3)	C34—C35	1.374 (4)
O6—C38	1.438 (4)	C34—H34	0.9300
O7—C40	1.350 (5)	C35—C36	1.372 (5)
O7—H7	0.8200	C35—H35	0.9300
O8—C50	1.233 (4)	C36—C37	1.375 (5)
O9—C52	1.361 (4)	C36—H36	0.9300
O9—C57	1.433 (4)	C37—H37	0.9300
C1—C2	1.391 (4)	C38—H38A	0.9600
C1—C10	1.422 (4)	C38—H38B	0.9600
C1—C11	1.455 (4)	C38—H38C	0.9600
C2—C3	1.409 (5)	C39—C40	1.375 (5)
C3—C4	1.335 (5)	C39—C48	1.420 (5)
C3—H3	0.9300	C39—C49	1.449 (5)
C4—C5	1.405 (5)	C40—C41	1.410 (6)
C4—H4A	0.9300	C41—C42	1.336 (6)
C5—C6	1.387 (6)	C41—H41	0.9300
C5—C10	1.426 (5)	C42—C43	1.414 (6)
C6—C7	1.354 (6)	C42—H42	0.9300
C6—H6	0.9300	C43—C44	1.393 (6)
C7—C8	1.416 (7)	C43—C48	1.416 (5)
C7—H7A	0.9300	C44—C45	1.352 (7)
C8—C9	1.370 (5)	C44—H44	0.9300
C8—H8	0.9300	C45—C46	1.396 (6)
C9—C10	1.413 (5)	C45—H45	0.9300
C9—H9	0.9300	C46—C47	1.368 (5)
C11—H11	0.9300	C46—H46	0.9300
C12—C13	1.492 (4)	C47—C48	1.394 (5)
C13—C14	1.390 (4)	C47—H47	0.9300
C13—C18	1.393 (4)	C49—H49	0.9300
C14—C15	1.382 (4)	C50—C51	1.482 (5)
C15—C16	1.376 (5)	C51—C56	1.381 (4)
C15—H15	0.9300	C51—C52	1.391 (5)
C16—C17	1.366 (5)	C52—C53	1.391 (5)
C16—H16	0.9300	C53—C54	1.372 (5)
C17—C18	1.357 (5)	C53—H53	0.9300
C17—H17	0.9300	C54—C55	1.373 (5)
C18—H18	0.9300	C54—H54	0.9300
C19—H19A	0.9600	C55—C56	1.374 (5)
C19—H19B	0.9600	C55—H55	0.9300
C19—H19C	0.9600	C56—H56	0.9300
C20—C21	1.389 (5)	C57—H57A	0.9600
C20—C29	1.432 (4)	C57—H57B	0.9600
C20—C30	1.438 (4)	C57—H57C	0.9600
			
C11—N1—N2	117.2 (3)	C28—C27—C26	120.3 (4)
C12—N2—N1	117.3 (3)	C28—C27—H27	119.8
C12—N2—H2	122 (2)	C26—C27—H27	119.8
N1—N2—H2	121 (2)	C27—C28—C29	122.0 (4)
C30—N3—N4	119.5 (3)	C27—C28—H28	119.0
C31—N4—N3	118.7 (3)	C29—C28—H28	119.0
C31—N4—H4B	120 (2)	C28—C29—C24	117.0 (4)
N3—N4—H4B	121 (2)	C28—C29—C20	123.2 (3)
C49—N5—N6	118.6 (3)	C24—C29—C20	119.8 (4)
C50—N6—N5	118.5 (3)	N3—C30—C20	120.1 (3)
C50—N6—H6A	116 (2)	N3—C30—H30	120.0
N5—N6—H6A	125 (2)	C20—C30—H30	120.0
C2—O1—H1	109.5	O5—C31—N4	119.9 (4)
C14—O3—C19	120.0 (3)	O5—C31—C32	121.7 (3)
C21—O4—H4	112 (3)	N4—C31—C32	118.3 (3)
C33—O6—C38	119.2 (3)	C37—C32—C33	117.6 (3)
C40—O7—H7	109.5	C37—C32—C31	115.2 (3)
C52—O9—C57	119.7 (3)	C33—C32—C31	127.1 (3)
C2—C1—C10	118.7 (3)	O6—C33—C34	122.2 (3)
C2—C1—C11	120.2 (3)	O6—C33—C32	117.2 (3)
C10—C1—C11	121.1 (3)	C34—C33—C32	120.6 (3)
O1—C2—C1	123.6 (3)	C35—C34—C33	120.6 (4)
O1—C2—C3	115.3 (4)	C35—C34—H34	119.7
C1—C2—C3	121.1 (4)	C33—C34—H34	119.7
C4—C3—C2	119.9 (4)	C36—C35—C34	119.6 (4)
C4—C3—H3	120.0	C36—C35—H35	120.2
C2—C3—H3	120.0	C34—C35—H35	120.2
C3—C4—C5	122.5 (4)	C35—C36—C37	120.2 (4)
C3—C4—H4A	118.7	C35—C36—H36	119.9
C5—C4—H4A	118.7	C37—C36—H36	119.9
C6—C5—C4	123.0 (5)	C36—C37—C32	121.4 (4)
C6—C5—C10	118.8 (5)	C36—C37—H37	119.3
C4—C5—C10	118.2 (4)	C32—C37—H37	119.3
C7—C6—C5	123.7 (6)	O6—C38—H38A	109.5
C7—C6—H6	118.2	O6—C38—H38B	109.5
C5—C6—H6	118.2	H38A—C38—H38B	109.5
C6—C7—C8	117.9 (5)	O6—C38—H38C	109.5
C6—C7—H7A	121.0	H38A—C38—H38C	109.5
C8—C7—H7A	121.0	H38B—C38—H38C	109.5
C9—C8—C7	120.7 (5)	C40—C39—C48	119.2 (4)
C9—C8—H8	119.7	C40—C39—C49	119.4 (4)
C7—C8—H8	119.7	C48—C39—C49	121.4 (4)
C8—C9—C10	121.3 (4)	O7—C40—C39	123.9 (4)
C8—C9—H9	119.4	O7—C40—C41	115.0 (5)
C10—C9—H9	119.4	C39—C40—C41	121.1 (5)
C9—C10—C1	122.7 (4)	C42—C41—C40	119.9 (6)
C9—C10—C5	117.7 (4)	C42—C41—H41	120.1
C1—C10—C5	119.6 (4)	C40—C41—H41	120.1
N1—C11—C1	119.9 (3)	C41—C42—C43	121.8 (6)
N1—C11—H11	120.1	C41—C42—H42	119.1
C1—C11—H11	120.1	C43—C42—H42	119.1
O2—C12—N2	121.6 (3)	C44—C43—C42	121.9 (6)
O2—C12—C13	121.1 (3)	C44—C43—C48	119.4 (6)
N2—C12—C13	117.3 (3)	C42—C43—C48	118.6 (5)
C14—C13—C18	117.4 (3)	C45—C44—C43	121.7 (6)
C14—C13—C12	127.2 (3)	C45—C44—H44	119.1
C18—C13—C12	115.4 (3)	C43—C44—H44	119.1
O3—C14—C15	122.0 (3)	C44—C45—C46	119.1 (6)
O3—C14—C13	117.2 (3)	C44—C45—H45	120.5
C15—C14—C13	120.8 (3)	C46—C45—H45	120.5
C16—C15—C14	119.6 (4)	C47—C46—C45	120.7 (5)
C16—C15—H15	120.2	C47—C46—H46	119.6
C14—C15—H15	120.2	C45—C46—H46	119.6
C17—C16—C15	120.5 (4)	C46—C47—C48	121.1 (4)
C17—C16—H16	119.8	C46—C47—H47	119.5
C15—C16—H16	119.8	C48—C47—H47	119.5
C18—C17—C16	119.8 (4)	C47—C48—C43	117.9 (5)
C18—C17—H17	120.1	C47—C48—C39	122.9 (4)
C16—C17—H17	120.1	C43—C48—C39	119.2 (5)
C17—C18—C13	121.9 (3)	N5—C49—C39	119.8 (4)
C17—C18—H18	119.0	N5—C49—H49	120.1
C13—C18—H18	119.0	C39—C49—H49	120.1
O3—C19—H19A	109.5	O8—C50—N6	119.8 (4)
O3—C19—H19B	109.5	O8—C50—C51	121.6 (4)
H19A—C19—H19B	109.5	N6—C50—C51	118.7 (4)
O3—C19—H19C	109.5	C56—C51—C52	118.2 (4)
H19A—C19—H19C	109.5	C56—C51—C50	115.6 (4)
H19B—C19—H19C	109.5	C52—C51—C50	126.2 (4)
C21—C20—C29	119.0 (4)	O9—C52—C53	122.1 (4)
C21—C20—C30	119.7 (4)	O9—C52—C51	117.6 (4)
C29—C20—C30	121.3 (3)	C53—C52—C51	120.3 (4)
O4—C21—C20	123.9 (4)	C54—C53—C52	119.6 (4)
O4—C21—C22	115.7 (4)	C54—C53—H53	120.2
C20—C21—C22	120.4 (4)	C52—C53—H53	120.2
C23—C22—C21	120.3 (4)	C53—C54—C55	120.9 (5)
C23—C22—H22	119.9	C53—C54—H54	119.6
C21—C22—H22	119.9	C55—C54—H54	119.6
C22—C23—C24	121.9 (4)	C54—C55—C56	119.2 (4)
C22—C23—H23	119.0	C54—C55—H55	120.4
C24—C23—H23	119.0	C56—C55—H55	120.4
C25—C24—C23	121.3 (5)	C55—C56—C51	121.8 (4)
C25—C24—C29	120.1 (4)	C55—C56—H56	119.1
C23—C24—C29	118.6 (4)	C51—C56—H56	119.1
C26—C25—C24	120.8 (5)	O9—C57—H57A	109.5
C26—C25—H25	119.6	O9—C57—H57B	109.5
C24—C25—H25	119.6	H57A—C57—H57B	109.5
C25—C26—C27	119.7 (5)	O9—C57—H57C	109.5
C25—C26—H26	120.1	H57A—C57—H57C	109.5
C27—C26—H26	120.1	H57B—C57—H57C	109.5

### Hydrogen-bond geometry (Å, °)

**Table:**

*D*—H···*A*	*D*—H	H···*A*	*D*···*A*	*D*—H···*A*
O1—H1···N1	0.82	1.84	2.556 (3)	145
N2—H2···O3	0.91 (1)	1.94 (2)	2.622 (3)	131 (3)
N2—H2···O8^i^	0.91 (1)	2.41 (2)	3.094 (4)	132 (2)
O4—H4···N3	0.86 (1)	1.82 (2)	2.538 (4)	141 (3)
N4—H4B···O6	0.91 (1)	1.95 (2)	2.647 (4)	132 (3)
O7—H7···N5	0.82	1.80	2.517 (4)	146
N6—H6A···O9	0.89 (1)	1.89 (2)	2.621 (4)	138 (3)
C11—H11···O8^i^	0.93	2.46	3.122 (4)	129
C38—H38B···O2^ii^	0.96	2.35	3.090 (5)	133
C57—H57B···O4^iii^	0.96	2.56	3.435 (5)	151

Symmetry codes: (i) *x*, *y*, *z*−1; (ii) −*x*+2, −*y*+1, −*z*+1; (iii) −*x*+1, −*y*+1, −*z*+1.
